# The effect of soluble E-selectin on tumor progression and metastasis

**DOI:** 10.1186/s12885-016-2366-2

**Published:** 2016-05-24

**Authors:** Shin-Ae Kang, Celine A. Blache, Sandra Bajana, Nafis Hasan, Mohamed Kamal, Yoshihiro Morita, Vineet Gupta, Bilegtsaikhan Tsolmon, Stephen K. Suh, David G. Gorenstein, Wajeeha Razaq, Hallgeir Rui, Takemi Tanaka

**Affiliations:** Department of Pathology, University of Oklahoma Health Sciences Center, 975 NE, 10th, Oklahoma City, OK 73104 USA; Thomas Jefferson University, Pharmaceutical Sciences, 1020 Locust St, Philadelphia, PA 19107 USA; John Theurer Cancer Center, Hackensack University Medical Center, Hackensack, NJ 07601 USA; Institute of Molecular Medicine, University of Texas Health Science Center at Houston, 1825 Hermann Pressler, Houston, TX 77030 USA; Department of Internal Medcine, Stephenson Cancer Center, University of Oklahoma Health Sciences Center, 800 NE, 10th, Oklahoma City, OK 73104 USA; Department of Pathology, Medical College of Wisconsin Cancer Center, 8701 Watertown Plank Rd, Milwaukee, WI 53226 USA; Stephenson Cancer Center at the University of Oklahoma Health Sciences Center, 975 NE 10th, Oklahoma City, OK 73104 USA

**Keywords:** sE-selectin, E-selectin, Shear-resistant adhesion, CD44, Hematogenous metastasis

## Abstract

**Background:**

Distant metastasis resulting from vascular dissemination of cancer cells is the primary cause of mortality from breast cancer. We have previously reported that E-selectin expression on the endothelial cell surface mediates shear-resistant adhesion and migration of circulating cancer cells via interaction with CD44. As a result of shedding, soluble E-selectin (sE-selectin) from the activated endothelium is present in the serum. In this study, we aimed to understand the role of sE-selectin in tumor progression and metastasis.

**Methods:**

We investigated the effect of sE-selectin on shear-resistant adhesion and migration of metastatic breast cancer cells and leukocytes in vitro and in vivo.

**Results:**

We found that sE-selectin promoted migration and shear-resistant adhesion of CD44^+^^/high^ breast cancer cell lines (MDA-MB-231 and MDA-MB-468) to non-activated human microvessel endothelial cells (ES-HMVECs), but not of CD44^-/low^ breast cancer cell lines (MCF-7 and T-47D). This endothelial E-selectin independent, sE-selectin-mediated shear-resistant adhesion was also observed in a leukocyte cell line (HL-60) as well as human peripheral blood mononuclear cells (PBMCs). Additionally, the incubation of MDA-MB-231 cells with sE-selectin triggered FAK phosphorylation and shear-resistant adhesion of sE-selectin-treated cells resulted in increased endothelial permeabilization. However, CD44 knockdown in MDA-MB-231 and HL-60 cells resulted in a significant reduction of sE-selectin-mediated shear-resistant adhesion to non-activated HMVECs, suggesting the involvement of CD44/FAK. Moreover, functional blockade of ICAM-1 in non-activated HMVECs resulted in a marked reduction of sE-selectin-mediated shear-resistant adhesion. Finally, the pre-incubation of CD44^+^ 4 T1 murine breast cancer cells with sE-selectin augmented infiltration into the lung in E-selectin K/O mice and infusion of human PBMCs pre-incubated with sE-selectin stimulated MDA-MB-231 xenografted breast tumor growth in NSG mice.

**Conclusions:**

Our data suggest that circulating sE-selectin stimulates a broad range of circulating cells via CD44 and mediates pleiotropic effects that promote migration and shear-resistant adhesion in an endothelial E-selectin independent fashion, in turn accelerating tissue infiltration of leukocytes and cancer cells.

**Electronic supplementary material:**

The online version of this article (doi:10.1186/s12885-016-2366-2) contains supplementary material, which is available to authorized users.

## Background

Tissue infiltration of circulating cells is critical for multiple aspects of tumor progression and metastasis. First, extravasation of circulating leukocytes into the primary tumor aids in the development of a tumor stroma that provides a fertile environment for cancer cells to survive and grow. Second, extravasation of circulating tumor cells into the tissue parenchyma of distant organs gives rise to metastasis. Despite the recognized importance of tissue infiltration in tumor progression and metastasis as well as pathologic alterations in the global serum protein profiles of cancer patients, it is uncertain whether circulating factors in the vascular space extrinsically stimulate circulating cells to infiltrate tissues. During tissue infiltration, inflamed vascular endothelium, characterized by expression of E-selectin and CAMs, functions as a gateway to tissue parenchyma. Tissue infiltration by circulating cells is governed by a multi-step adhesion cascade, mediated by sequential interactions and crosstalk between adhesion molecules expressed on the endothelial cell surface and their counter-receptor ligands expressed on circulating cells [1–4]. A robust interaction is required for this attachment to resist swift blood flow. Weak interaction prevents effective attachment of the circulating cells to the endothelial surface. The initial adhesion of circulating cells subsequently leads to firm attachment that opens the endothelial junction enabling transendothelial migration into tissue parenchyma toward cytokine or chemokine stimuli [5, 6].

Adhesion molecules, such as selectins, ICAM, and VCAM, expressed on the endothelial surface in response to inflammatory stimuli are the primary mediators of tissue infiltration [7, 8]. E-selectin (CD62E, ELAM-1, or LECAM-2) is expressed exclusively on the luminal surfaces of inflamed vasculature [9] and mediates rolling and vascular adhesion through interaction with carbohydrate ligands [10, 11]. A variety of ligands for E-selectin have been identified in different types of cells; these include CD44 (HCELL) [12–14], Mac-2 binding protein [15], Muc-1 [16], death receptor-3 [17], and ESL-1 [18]. The expression of E-selectin is temporally and spatially limited. E-selectin is shed into circulation (soluble E-selectin: sE-selectin) or internalized rapidly after activation. An abundance of sE-selectin in the serum is indicative of inflammation [19–23], and circulating sE-selectin levels are consistently high among individuals with chronic inflammation compared to their healthy counterparts [24]. Similarly, elevated serum levels of sE-selectin were reported and positively associated with tumor grade, tumor stage, and metastases in breast cancer [3, 25]. Despite the positive associations between sE-selectin and breast cancer progression and metastasis, it is unclear whether sE-selectin plays any functional role in tumor progression. In the present study, we demonstrate that sE-selectin functions as a signaling molecule in circulation with the ability to 1) enhance shear-resistant adhesion and migration of CD44^+/high^ breast cancer cells (BCs) and leukocytes in an endothelial E-selectin independent fashion, 2) activate focal adhesion kinase (FAK), 3) promote homing of CD44^+/high^ BCs to the lung, and 4) stimulate tumor growth and intratumoral infiltration of PBMCs. These findings identify a possible extrinsic mechanism by which an inflammation-associated factor (i.e., sE-selectin) present in circulation pleiotropically stimulates circulating tumor cells toward tumor progression and metastasis.

## Methods

### Cell lines and culture conditions

Human breast cancer cell lines MDA-MB-231, MDA-MB-468, T-47D, and MCF-7, human leukocyte cell line HL-60, and murine breast cancer cell line 4 T1 were purchased from ATCC (Rockville, MD). MDA-MB-231, MDA-MB-468, MCF-7, and 4 T1 cells were cultured in Dulbecco’s Modified Eagle Medium (DMEM; Cellgro) supplemented with 10 % fetal bovine serum (FBS), 2 mM GlutaMAX, and 100U/ml Penicillin-Streptomycin (Life Technology, Grand Island, NY). T-47D and human PBMCs cells were cultured in RPMI-1640 supplemented with 10 % FBS, 2 mM GlutaMAX, and 100U/ml Penicillin-Streptomycin. HL-60 cells were cultured in IMDM supplemented with 20 % FBS, 100U/ml Penicillin-Streptomycin, and Insulin-Transferrin-Selenium (100 mg/L Insulin; 5.5 mg/L Transferrin; 6.7 μg/L Selenium; Corning, Manassas, VA). CD44 knockdown clones were generated by transducing the cells with CD44 shRNA lentivirus or control shRNA (Santa Cruz, CA), followed by selection with puromycin. 4 T1-Luc was generated by transducing 4 T1 cells with luciferase lentivirus (Capital Biosciences) followed by selection using 6 μg/ml puromycin (InvivoGen). Human Microvascular Endothelial cells (HMVECs) and E-selectin-expressing Tet-on inducible HMVECs [26] were cultured in endothelial basal medium-2 (EBM-2; Lonza) supplemented with 2 % tet-approved FBS and an EGM SingleQuot Kit containing epidermal growth factor (EGF), hydrocortisone, gentamicin, and amphotericin B (GA-1000; Lonza). All cells were cultured in 5 % CO_2_ humid chambers at 37 °C.

### Reagents and antibodies

Recombinant human sE-selectin was purchased from R&D Systems. Monoclonal antibodies for pFAK and total FAK were purchased from Cell Signaling. Blocking antibodies for ICAM and VCAM were purchased from Santa Cruz and Biolegend, respectively. FAK inhibitor II was purchased from Calbiochem.

### In vitro cell adhesion assay

HMVECs were grown to confluence in the flow chamber (μ-Slide I 0.4 Luer for cancer cells and 0.2 for leukocytes; Ibidi, Madison, WI) coated with collagen I and fibronectin. The cells (10^5^ cells/ml) were pre-incubated with 100 nM sE-selectin for 10 min and perfused over a 5 min period into the flow chamber in 1 % FBS containing DMEM medium at a shear stress rate of 1 dyn/cm^2^. Unbound cells were washed off with DMEM containing 1 % FBS. Using a light microscope at a final magnification of 100×, the number of cells that adhered to endothelial cells was determined by counting cells in at least five random fields. The data are expressed as the mean of triplicate experiments.

### In vitro permeability assay

HMVECs were grown to confluence on the top-facing side of a collagen-coated 0.4-μm-pore transwell chamber (Corning). MDA-MB-231 cells were pre-incubated with 100 nM sE-selectin for 10 min at 37 °C. MDA-MB-231 cells (2 × 10^3^ cells) suspended in EBM-2 media and 2000-kDa FITC-dextran (final concentration of 1 mg/ml, Sigma-Aldrich) were seeded into the upper chamber, and 600 μl EBM-2 media was added to lower chamber. Then, at the indicated time points, 50-μl aliquots were removed from the lower chamber and the fluorescence was measured at 485/530 nm with a CLARIOstar fluorimeter (BMG Labtech).

### Cell migration assay

Transwell migration assays were performed using a 12.0-μm or 5.0-μm pore transwell chamber (Becton Dickinson). Cancer cells (2.5 × 10^4^ cells) or leukocytes (1.0 × 10^5^ cells) suspended in serum-free media were applied to the upper chamber and 100 nM sE-selectin was added to the lower chamber. After overnight incubation at 37 °C, migrated cells were stained using a HEMA 3 staining kit (Pierce) and counted under a light microscope.

### Anoikis assay

Anoikis resistance was measured using ultra-low attachment plates (Corning). Cancer cells (10^4^ cells) were suspended in 1 % FBS containing medium with or without sE-selectin, seeded into 96-well ultra-low attachment plates, and incubated for 72 h. The number of viable and non-viable cells was determined by trypan blue exclusion using a hemocytometer.

### Animal models

All animal housing and handling procedures were in accordance with institutional guidelines at the University of Oklahoma and Thomas Jefferson University. Six-week-old female Balb/C mice (Jackson Laboratory) or E-selectin knockout mice (C.129S4-*Sele*^*tm1Dmil*^/J; Jackson Laboratory) were used for the in vivo cell adhesion assay. Female Balb/C mice or E-selectin knockout mice (*n* = 3) were injected intravenously with 4 T1-luc murine breast cancer cells (3 × 10^4^ in 100 μl saline) via tail vein. Cancer cells were pre-incubated with 100 nM sE-selectin for 10 min at 37 °C and washed with PBS to remove sE-selectin carry over before injection. Seven days later, whole lungs were harvested and total RNA was isolated using TRIzol (Life Technologies). Total RNA was reverse transcribed using random hexamer primers and SuperScript® III First-Strand Synthesis Supermix (Life Technologies). qPCR reactions (80 cycle) were performed using TaqMan gene expression master mix (Life Technologies) and the Biorad CFX96™ Touch Real-Time PCR Detection System (Biorad). Luciferase and mouse GAPDH primers were used (Life Technologies). Data are presented as delta-Ct relative to an endogenous control, GAPDH. For leukocyte infiltration, MDA-MB-231 breast cancer cells were injected into the mammary fat pads of 7-week-old female NOD SCID IL2 receptor gamma chain knockout mice (NSG mice: NOD.Cg-Prkdc^scid^ Il2rg^tm1Wjl^/SzJ; Jackson Laboratory, Bar Harbor, ME). Freshly isolated PBMCs from healthy donors were incubated with 10 μM Calcein AM (BD bioscience, San Jose, CA) for 30 min. After brief a wash, the Calcein positive PBMCs (2 × 10^7^) were incubated with sE-selectin or saline and slowly infused over 1 min via tail vein into NSG mice bearing tumors of approximately 300 mm^3^(LxWxH). Tumors were harvested to analyze the tissue migration of Calcein-labeled PBMCs. For tumor growth, MDA-MB-231-TGL cell line (a kind gift from Dr. Massague, Memorial Sloan-Kettering Cancer Center) was used. Human PBMCs (1 × 10^7^ cells) that were treated with or without sE-selectin were infused via tail vein once a week. Tumor growth was monitored by measuring bioluminescence using IVIS Lumina XR (Caliper, MA) once a week by intraorbital injection of 100 μl luciferase (15 μg/ml).

### Flow cytometry

Human CD44-APC (IM7) conjugated antibody (BD Biosciences, CA) was used to select shRNA-infected cells using a Coulter MoFlo cell sorter (Beckman Coulter, Brea, CA). For phenotypic analysis of human PBMCs, tissue was homogenized in cold RPMI, filtered through a 50-μm cell strainer, and washed in PBS. The single cell homogenates were incubated with conjugated antibodies (CD4-APCeFluor780, CD45-FITC, CD8-PerCPCy5.5, CD14-PE, and CD11c-APC) for 20 min at 4 °C. Cells were analyzed on an LSRII flow cytometer (BD Bioscience). All data were analyzed with FlowJo software.

### Statistical analysis

All experiments were carried out in triplicate and repeated at least twice independently. The results are presented as Mean ± *SD*. Comparisons between groups were analyzed using Student’s *t*-test. Statistical significance is expressed as *p < 0.05; **p < 0.01; ***p < 0.001.

## Results

### Soluble E-selectin enhances the shear-resistant adhesion, migration, and anoikis-resistant survival of CD44^+/high^ breast cancer cells

**Fig. 1:**
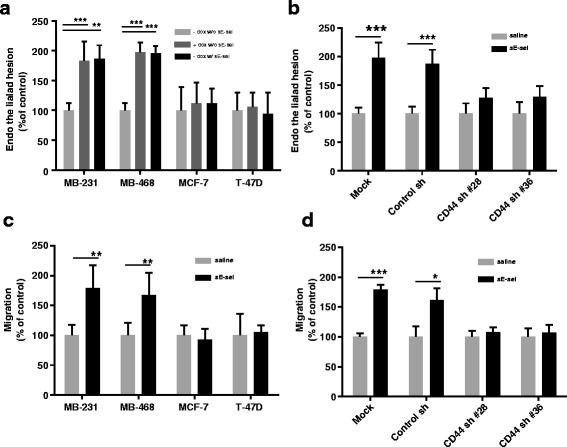
Soluble E-selectin induces adhesion and migration of CD44^+/high^ BCs: **a** Effect of sE-selectin on shear resistant adhesion of BCs to endothelial cells. A monolayer of ES-HMVECs was grown on the flow chamber. For endothelial E-selectin expression, ES-HMVECs were incubated with or without doxycycline (1500 ng/ml) for 4 h. BCs suspensions were incubated with 100 nM sE-selectin for 10 min and then were infused into flow chambers. The shear-resistant adhesion of BC cell lines (10^5 ^cells/ml) was tested at 37 °C for 5 min at a rate of 1 dyn/cm^2^. BCs adhering to endothelial cells were counted under a light microscope. Data were expressed as percentage of control (-Dox/-sEselectin as 100 %). **b** Effect of CD44 knockdown of MDA-MB-231 cells on sE-selectin-induced shear-resistant adhesion. Suspensions of MDA-MB-231 cells with CD44 shRNA or control shRNA clone (10^5^ cells/ml) were treated with sE-selectin or saline and infused into flow chambers after a brief wash. The adhesion of the cells to non-activated ES-HMVECs (-Dox) were counted under a light microscope. Data were expressed as percentage of control (saline treated as 100 %). **c** Effect of sE-selectin on the migration of BCs. BCs were seeded into the upper chambers (12-μm pore), soluble E-selectin (100 nM) was added to the lower chambers, and plates were incubated overnight at 37 °C. Migrated cells were stained using an HEMA3 staining kit and were counted under light microscopy. Data were expressed as percentage of control (saline treated as 100 %). **d** Effect of CD44 knockdown of MDA-MB-231 cells on sE-selectin-induced migration. CD44 knockdown clones of MDA-MB-231 cells or control shRNA were added to the upper chambers and migrated cells were counted after overnight incubation at 37 °C. The data represent Mean ± S.D. *p < 0.05; **p < 0.01; ***p < 0.001 *vs*. saline, Student’s *t*-test. Data were summarized from an experiment conducted in triplicate and repeated twice

To investigate the effect of sE-selectin on factors pertinent to hematogenous metastasis, we evaluated shear-resistant adhesion, migration, and anoikis-resistant survival using two estrogen receptor (ER)-negative/CD44^+/high^ BCs (MDA-MB-231 and MDA-MB-468), two ER-positive/CD44^-/low^ BCs (T-47D and MCF-7), and a line of Tet-on inducible, E-selectin expressing, human microvessel endothelial cells (ES-HMVECs) [26]. A confluent monolayer of ES-HMVECs was grown on a collagen/fibronectin-coated parallel flow chamber and was incubated with or without doxycycline for 4 h to induce the expression of E-selectin. A single cell suspension of BCs was infused into a chamber to recapitulate the shear-resistant adhesion of disseminated/circulating tumor cells to vascular endothelial cells. The adherent BCs were counted. Incubation of MDA-MB-231 and MDA-MB-468 BCs with sE-selectin increased the shear-resistant adhesion to doxycycline-untreated ES-HMVECs (i.e., absence of E-selectin expression on ES-HMVECs) 1.9 fold (*p* < 0.05) and 2.0 fold (*p* < 0.001), respectively, compared with adhesion of saline-treated BCs (Fig. 1a). The adhesion of saline-treated MDA-MB-231 and MDA-MB-468 BCs to doxycycline-untreated ES-HMVECs was negligible, but increased significantly when E-selectin expression was induced in ES-HMVECs by doxycycline (1.8 fold and 2.0 fold, respectively, *p* < 0.001). The extent of shear-resistant adhesion of sE-selectin-treated MDA-MB-231 and MDA-MB-468 BCs to untreated ES-HMVECs was similar to that of saline-treated BCs to activated ES-HMVECs. In contrast, neither sE-selectin nor endothelial E-selectin enhanced the shear-resistant adhesion of ER+/CD44^-/low^ MCF-7 and T47D BCs. These results suggest the involvement of CD44 in sE-selectin-mediated adhesion. Previously, we demonstrated a physical interaction between E-selectin and CD44 isolated from MDA-MB-231 BCs [13]; we then examined the effect of sE-selectin in shRNA-mediated CD44-knockdown MDA-MB-231 BCs. Soluble E-selectin-induced shear-resistant adhesion was significantly reduced in two CD44 knockdown clones of MDA-MB-231 BCs (clone 28, 0.29 fold, and clone 36, 0.30 fold, *p* < 0.001) as compared with saline controls (Fig. 1b).

To further examine the role of sE-selectin on migration, we performed a Boyden chamber migration assay. BCs were added to the upper chamber, while the lower chamber contained culture media, with or without sE-selectin. The presence of sE-selectin enhanced migration of CD44^+/high^ MDA-MB-231 BCs (1.8 fold, *p* = 0.006) and MDA-MB-468 BCs (1.7 fold, *p* = 005) (Fig. 1c). However, sE-selectin did not affect the migration of CD44^-/low^ MCF-7 BCs or T-47D BCs (Fig. 1c). Soluble E-selectin-induced migration was also significantly reduced in CD44 knockdown clones of MDA-MB-231 BCs (Fig. 1d). Moreover, sE-selectin increased anoikis resistance in MDA-MB-231 BCs (1.36 fold, *p* < 0.001) and MDA-MB-468 BCs (1.16 fold, *p* = 0.02), but this effect was not observed in MCF-7 and T-47D BCs (Additional file 1: Figure S1). Therefore, these data suggest that sE-selectin specifically enhances anoikis resistant survival, shear resistant adhesion, and migration of CD44^+/high^ BCs.

### Soluble E-selectin enhances shear-resistant adhesion and migration in leukocytes

**Fig. 2:**
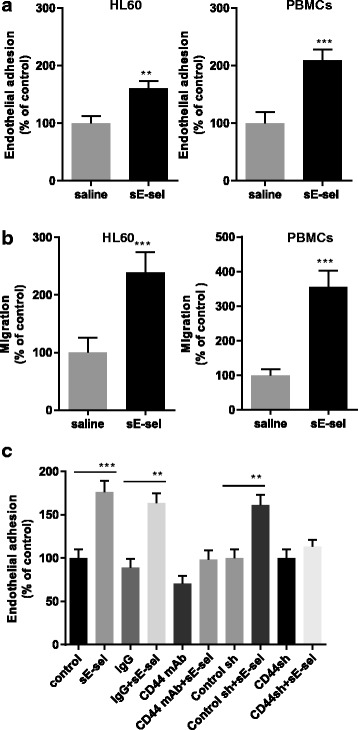
Soluble E-selectin enhances migration of human leukocytes in vitro. **a** Effect of sE-selectin on the adhesion of leukocytes to non-activated p-HMVECs. A monolayer of p-HMVECs was grown on the parallel flow chamber. The cells (10^5^ cells/ml) were incubated with 100 nM sE-selectin for 30 min and then infused into the chamber at 37 °C over a 5 min period at a shear stress rate of 1 dyn/cm^2^. Adhesion was expressed as a percent relative to adhesion of saline-treated control cells. **b** Migration assay was performed using Boyden chamber. HL-60 cells (5 × 10^5^ cells) were seeded in upper chambers (5-μm pores) and 100 nM sE-selectin was supplemented to the lower chambers. The number of migrated cells was counted after 16 h. Migration is expressed as a percent relative to saline-treated control. **c** Effect of CD44 knockdown of HL-60 on sE-selectin-induced adhesion. The data represent Mean ± S.D. *p < 0.05; **p < 0.01; ***p < 0.001, Student’s *t* test. Data were summarized as % of respective control from an experiment conducted in triplicate and repeated twice

To further assess the effect of sE-selectin on circulating cells, leukocyte cell line HL-60 and human peripheral blood mononuclear cells (PBMCs) were used in adhesion and migration assays. Leukocytes (10^5^ cells/ml) were infused into a confluent monolayer of parental HMVECs (p-HMVECs) grown on a collagen/fibronectin-coated parallel flow chamber at 1 dyn/cm^2^ for 5 min and the adherent cells were counted. Consistent with the data from analysis of BCs, sE-selectin enhanced the shear-resistant adhesion of HL-60 (1.6 fold, *p* < 0.001) and PBMCs (2.1 fold, *p* < 0.001) to non-activated pHMVEC (Fig. 2a). Additionally, sE-selectin enhanced the migration of HL-60 (2.4 fold, *p* < 0.001), and PBMCs (3.6 fold, *p* < 0.001) (Fig. 2b). Functional blockade of CD44 in HL-60 using CD44 blocking antibody or a knockdown resulted in reductions of sE-selectin-mediated adhesion to non-activated p-HMVECs (*p* < 0.001). Taken together, these results indicate that sE-selectin may have biological function via CD44 in a broad range of circulating cells including leukocytes and cancer cells.

### The shear-resistant adhesion of sE-selectin-treated MDA-MB-231 BCs increases the permeability of non-activated p-HMVECs

**Fig. 3:**
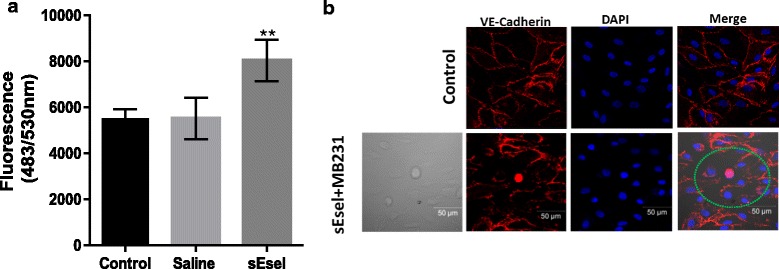
Incubation of breast cancer cells with sE-selectin increases permeability of p-HMVECs: **a** Effect of adhesion of sE-selectin-treated BC on endothelial permeability. HMVECs were grown to confluence onto collagen-coated 0.4-μm-pore transwell chambers. MDA-MB-231 cells were pre-incubated with sE-selectin or saline for 10 min and plated onto the upper chamber with 2000-kDa FITC-dextran. The same amounts of FITC dextran was added to the control well without addition of MDA-MB-231 cells. The fluorescence in the lower chamber as a result of endothelial permeability was measured at 60 min after the addition of cells and dye. The data represent Mean ± SD. Statistical significance was determined by Student’s *t*-test (saline treated-MDA-MB-231 *vs*. sE-selectin-treated MDA-MB-231). **b** Endothelial gap formation following adhesion of sE-selectin-treated MDA-MB-231 cells. MDA-MB-231 cells treated with sE-selectin and infused into flow chambers over a 5 min period at a shear stress rate of 1 dyn/cm^2^. Cells were fixed for 10 min, immunostained with VE-cadherin (*red*), and counter-stained with DAPI (*blue*). The image was visualized by confocal microscope. Control indicates addition of no cells

While leaky endothelium is a hallmark of tumor-associated vasculatures, under physiologic conditions, endothelial cells are connected through tight junctions. Adhesion of cancer cells to the activated endothelium is reported to induce endothelial permeability for subsequent transendothelial migration [27, 28]. We next evaluated whether the shear-resistant adhesion of sE-selectin-treated MDA-MB-231 BCs enhanced the permeability of non-activated p-HMVECs. A single cell suspension of MDA-MB-231 BCs was pre-incubated with sE-selectin or saline. After a brief spin followed by washing with PBS, the cells were layered onto a confluent monolayer of non-activated p-HMVECs with 2000 kDa FITC-dextran as a fluorescent tracer. Following the addition of sE-selectin-treated MDA-MB-231 cells, a steep increase in fluorescent leakage through the HMVEC monolayer was observed (Fig. 3a). In contrast, the adhesion of saline-treated MDA-MB-231 BCs to non-activated p-HMVECs did not have a significant effect on endothelial permeabilization over the course of the 60 min assay (Fig. 3a). Incubation of p-HMVECs with sE-selectin alone had no effect on endothelial permeability (data not shown). To confirm increased endothelial permeability, the endothelial junction was immunostained after the adhesion of BCs to non-activated p-HMVECs. A single cell suspension of MDA-MB-231 BCs was treated with sE-selectin and then washed with PBS to remove sE-selectin. The cells were infused into a flow chamber at a rate of 1 dyn/cm^2^ for 5 min. After a brief wash, the cells were fixed for immunostaining with VE-cadherin. The shear-resistant adhesion of sE-selectin-treated MDA-MB-231 BCs to non-activated p-HMVECs resulted in the appearance of a visible gap and disappearance of VE-cadherin surface expression (Fig. 3b). These data suggest that the adhesion of sE-selectin treated CD44^+/high^ BCs permeabilizes non-activated endothelium.

### Soluble E-selectin activates focal adhesion kinase

**Fig. 4:**
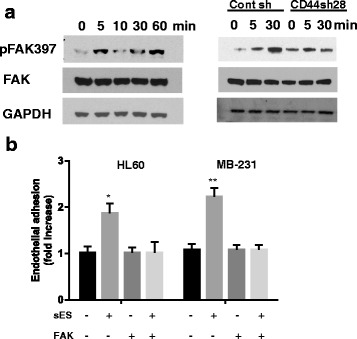
Soluble E-selectin triggers FAK activation: **a** Activation of FAK by sE-selectin in MDA-MB-231. An MDA-MB-231 suspension was incubated with sE-selectin (100 nM) for the indicated period of time (0-60 min). CD44 shRNA knockdown clones or control shRNA of MDA-MB-231 cells (clone 28) were incubated with sE-selectin for the indicated time. Cell lysate (30 μg) was separated by SDS-PAGE and probed with pFAK (Y397) and total FAK, followed by HRP-conjugated secondary antibody. GAPDH was used as a loading control. The blot was visualized by ECL. **b** FAK inhibitor blocks sE-selectin-mediated adhesion. MDA-MB-231 or HL-60 cells were co-incubated with vehicle alone, sE-selectin, FAK inhibitor II (5 μM), or sE-selectin + FAK inhibitor II for 30 min in a rotation wheel. After a brief wash with PBS, the cells were infused into parallel flow chambers over a 5 min period at a shear stress rate of 1 dyn/cm^2^. The adherent cells were counted under a microscope. The data were summarized as fold increase of adhesion of MDA-MB-231 and HL60 cells. The data represent Mean ± SD. Statistical significance was determined by Student’s *t*-test

To determine the underlying mechanism of sE-selectin-mediated adhesion and migration, a single cell suspension of MDA-MB-231 BCs was incubated with sE-selectin for 5 to 60 min. Soluble E-selectin triggered the phosphorylation of focal adhesion kinase (FAK) at Tyr-397 after 5 min of incubation; whereas there were no changes in total FAK expression (Fig. 4a). However, sE-selectin did not affect FAK phosphorylation in the CD44sh 28 clone (Fig. 4a), suggesting the involvement of CD44 in sE-selectin-mediated FAK phosphorylation. To confirm the involvement of FAK signaling in sE-selectin-mediated effects, adhesion assays were performed using pathway-specific inhibitors at a concentration that did not affect cell viability (Additional file 1: Figure S2). Suspensions of MDA-MB-231 or HL-60 cells were co-incubated with sE-selectin and FAK inhibitor (FAK inhibitor II, 5 μM) for 30 min. After a brief spin followed by washing with PBS, the cells were then infused into parallel flow chambers. FAK inhibitor II abolished sE-selectin-mediated shear-resistant adhesion to non-activated HMVECs (Fig. 4b).

### Pre-activation of 4 T1 BCs with sE-selectin results in enhanced homing in vivo

**Fig. 5:**
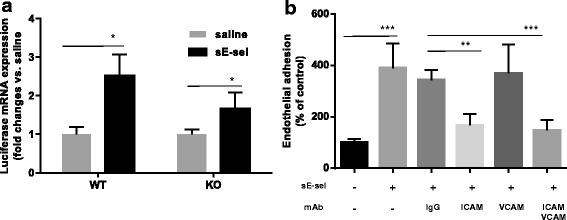
Soluble E-selectin enhances homing of 4T1 cells to the lung in mice: **a** Effect of sE-selectin on infiltration of 4 T1 cells into the lung. Luciferase-expressing 4 T1 murine breast cancer cells were pre-incubated with sE-selectin (100 nM) and, following washing with PBS to eliminate sE-selectin carryover, were intravenously injected into female Balb/C or E-selectin knockout mice. Seven days later, lungs were harvested and luciferase mRNA was analyzed by qRT-PCR and normalized by GAPDH. The data represent Mean ± SD. Statistical significance was determined by Student’s *t*-test. **b** sE-selectin-mediates adhesion is ICAM-1 dependent. Parental-HMVECs were pre-incubated with monoclonal antibody (1 μg in 100 μl) against ICAM-1 or VCAM-1 or the same amount of normal IgG for 1 h at 37 °C prior to the adhesion assay. After a brief wash, MDA-MB-231 BCs that were treated with sE-selectin for 30 min were infused into parallel flow chambers. The data represent Mean ± S.D. **p < 0.01; ***p < 0.001 *vs*. -/-, Student’s *t* test. Data were summarized as % of control (-/-) from an experiment conducted in triplicate, and repeated twice

To validate the effect of sE-selectin on hematogenous metastasis, we used a mouse model of forced lung metastasis. Murine 4 T1 BCs expressing luciferase (4T1-Luc) were pre-incubated with mouse sE-selectin and injected into wild-type Balb/C or E-selectin knockout mice via tail vein. Seven days after the injection, the mice were perfused with warm saline to remove residual cells in the circulation. Relative luciferase mRNA expression was measured in the lung. Pre-incubation of 4 T1 cells with sE-selectin increased homing to the lung by 2.5 fold (*p* = 0.03) in wild-type mice and 1.7 fold (*p* = 0.04) in E-selectin knockout mice compared with saline-treated 4 T1-Luc (Fig. 5a). The increased extent of lung homing in wild-type mice may be a consequence of endogenous E-selectin expression on the vessel or sE-selectin shed after activation in wild-type mice. To identify cellular adhesion molecules (CAMs) that support endothelial E-selectin-independent/sE-selectin-mediated shear-resistant adhesion, functional blocking antibodies against ICAM or VCAM were used to pretreat non-activated p-HMVECs for 1 h prior to the flow adhesion assay. The pre-incubation of non-activated p-HMVECs with ICAM-1 blocking antibody resulted in a significant reduction of sE-selectin-mediated shear-resistant adhesion; whereas, VCAM-1 blockade did not affect sE-selectin-mediated shear-resistant adhesion (Fig. 5b).

### Pre-activation of human PBMCs with sE-selectin stimulates tumor growth

**Fig. 6:**
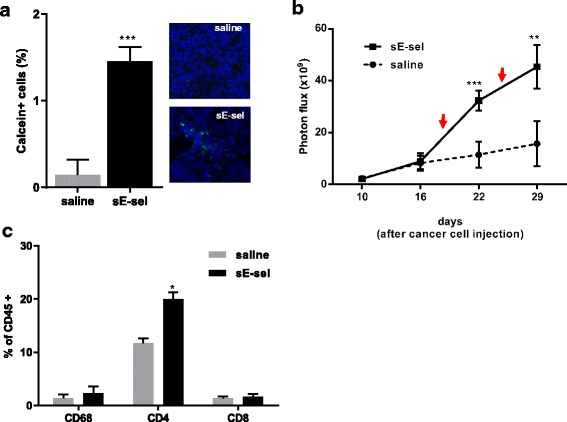
Soluble E-selectin enhances infiltration of PBMCs into tumor and tumor growth in NSG mice: **a** Human PBMC infiltration into breast tumor. Freshly isolated PBMCs were labeled with Calcein AM, followed by incubation with 100 nM sE-selectin for 15 min. After a brief washing to eliminate sE-selectin carryover, PBMCs (2 × 10^7^ cells) were infused into NSG mice bearing orthotopic breast tumors derived from MDA-MB-231 breast cancer cells (*n* = 4). Tumors were isolated 4 days after PBMC infusion and the numbers of Calcein-positive cells were counted by FACS. The histogram represents Mean ± SE (*n* = 4 mice). Frozen sections (5 μm) were stained with Hoechst 33342 (*blue*) to label nuclei. **b** Tumor growth following PBMC infusion. MDA-MB-231-luc cells was injected into mammary fat pad of NSG mice at day 0 and tumor growth was measured at day 10 and 16 to confirm similar growth rate between two groups. On day 18 and 25, human PBMCs (10^7^ cells) were infused via tail vein (*red arrow*) and tumor growth after the infusion was measured on day 22 and 29. Tumor growth was measured using bioluminescent imaging. The graph indicates photon flux from mice infused with PBMCs pre-incubated with saline (dashed line) or 100 nM sE-selectin (*solid line*). The data represent Mean ± SE (*n* = 5). **c** FACS analysis of human CD45+ leukocyte populations in the tumor. Tumors were harvested and single cell suspensions were labeled with human CD45, CD4, CD8, and CD68, and were analyzed by flow cytometry

To confirm pleiotropic effects of sE-selectin, we next explored the biological consequences of leukocyte activation by sE-selectin on tumor growth in vivo. Human PBMCs were freshly isolated from healthy donors and labeled with Calcein AM, then pre-treated with sE-selectin for 30 min at 37 °C. The PBMCs were infused via tail vein into NSG mice bearing human breast tumors derived from MDA-MB-231. Infiltration of Calcein positive PBMCs was measured by fluorescence-activated cell sorting (FACS) and visualized by fluorescent microscopy. Following a single intravenous infusion of sE-selectin-treated PBMCs, 1.45 ± 0.08 % of tumor cells were Calcein-positive, whereas, only 0.14 ± 0.1 % of tumor cells were Calcein-positive in mice receiving saline-treated PBMCs. This amounts to a ten-fold increase in PBMC infiltration when PBMCs are exposed to sE-selectin prior to infusion (*p* < 0.001) (Fig. 6a). Fluorescence microscopic analysis further confirmed that pre-incubation of PBMCs with sE-selectin resulted in an increased infiltration of Calcein-positive PBMCs into tumors, compared with tumor from mice infused with saline-treated PBMCs (Fig. 6a). Next, we evaluated whether the infiltration of sE-selectin-treated PBMCs into the tumor affected tumor growth. PBMCs treated with or without sE-selectin were infused at day 18 and 25 into NSG mice bearing MDA-MB-231 breast tumors carrying the luciferase gene (*n* = 5). Tumor growth was measured weekly at day 10, 16, 22 and 29 by bioluminescence. PBMCs were infused, and on day 22 (4 days post- PBMC infusion), we found that infusion of sE-selectin-treated PBMCs had increased tumor growth by 3.6 fold when compared to day 16 (prior to infusion). In contrast, in the mice receiving saline-treated PBMCs, only 1.4 fold tumor growth was noted. The differences in tumor growth rates between these groups were significant at four days after infusion (2.6 fold, *p* < 0.05). This trend remained the same at 29 days (Fig. 6b). FACS analysis of tumors further confirmed the presence of human CD45+ cells after two consecutive infusions of human PBMCs (Fig. 6c). Infusion of sE-selectin treated of PBMCs increased intratumoral infiltration by human CD4+ T-lymphocytes (20.2 %) significantly more than infusion of saline treated control PBMCs (11.5 %), but did not affect infiltration of CD8+ T-lymphocytes or CD68+ cells. Of note, the CD4/CD8 ratios of PBMCs used in this study were almost the same (CD4: 39.1, CD8: 37.2; Additional file 1: Figure S3). These data suggest that sE-selectin is a biologically functional signaling molecule in circulation after shedding and enhances the shear-resistant adhesion and migration of a broad range of CD44+ circulating cells (metastatic cancer cells or leukocytes), which additively promote disease progression via stromal development and homing of metastatic BCs to the lung.

## Discussion

Circulating E-selectin, sE-selectin, is a byproduct of endothelial E-selectin that originates from the external domain of E-selectin expressed on activated endothelium as a result of enzymatic cleavage or shedding from damaged or activated endothelial cells [29]. Several studies have reported chemotactic effects of sE-selectin in different cell types, including endothelial cells and leukocytes [30–32]. However, the effects of sE-selectin in tumor progression and metastasis is largely unknown. We and others have previously reported that the engagement of CD44 with endothelial E-selectin mediates shear-resistant adhesion of BCs and leukocytes to endothelial cells [13, 14, 33]. In addition to describing the roles of endothelial E-selectin, we found that sE-selectin functions as a circulating signaling molecule that potentiates shear-resistant adhesion and migration of CD44^+/high^ BCs and leukocytes in an endothelial E-selectin independent fashion and facilitates tumor progression and metastasis.

The adhesion cascade is governed by cell-cell interactions between circulating cells and endothelial cells via a sequential affinity interaction between adhesion molecules and the counter-receptor ligand. Thus, the ablation of adhesion molecules, such as selectins, ICAM-1, or VCAM-1, significantly impairs leukocyte infiltration. Given the E-selectin’s integral role in tissue infiltration as the first step mediator of adhesion, endothelial E-selectin-independent homing and shear-resistant adhesion of BCs and leukocytes were unexpected. Our data demonstrate that sE-selectin-mediated activation of the CD44/FAK axis of BCs enables ICAM-1-dependent adhesion at low shear stress, bypassing the requirement for engagement with endothelial E-selectin. A bypass of initial adhesion via endothelial E-selectin may occur when abundant CD44^+^ cells and circulating sE-selectin encountered endothelium expressing ICAM-1 at low shear stress. Similar bypass mechanism was reported by Thankamony and Sackstein as a novel paradigm of cell migration termed the “step 2-bypass pathway’”. The step 2-bypass pathway permits transendothelial migration of mesenchymal stem cells through interaction of endothelial VCAM-1 with activated VLA-4 without engagement of the chemokine receptors (CXCR4 and SDF-1) [34]. These data suggest the presence of alternative adhesion mechanisms that are supported by CAMs. In fact, the expression of CAMs is primarily induced by inflammatory cytokines; however, low levels of constitutive expression occurs in some organs and endothelial cell lines [35–37]. Additionally, double knockout of ICAM/VCAM is lethal [38, 39], while triple knockout of selectins (E-, P-, and L-selectin) does not completely ablate tissue infiltration of leukocytes to wounded areas [40]. These collectively indicate the presence of a selectin-independent alternative adhesion, which may be compensated by constitutive CAMs expression. This poses the question of whether individuals with breast cancer and synchronous chronic inflammation who have elevated sE-selectin and CD44+ circulating tumor cells (CTCs) are predisposed to increased risk of organ metastasis. Our data suggested that serum sE-selectin levels were associated with tumor size (Additional file 1: Figure S4). Additionally, several studies demonstrated positive correlations between high sE-selectin levels and worse clinical outcome [25, 41–43]. Sheen-Chen et al., reported that an elevated level of sE-selectin is significantly associated with advanced tumor size, lymph node involvement, metastasis, high histology grade, and reduced overall survival in breast cancer. The study further indicated that women with ER-negative breast tumors had a significantly higher level of sE-selectin than those with ER-positive breast tumors [41]. Similarly to cancer, elevated sE-selectin was also noted in individuals with chronic inflammation disorders including arthritis [44], diabetes [45], atherosclerosis [46], and alcoholism [47]. Our data demonstrated cellular responses to sE-selectin were concentration dependent and reached a plateau at 100 nM (Additional file 1: Figure S5); therefore, we used 100 nM sE-selectin in this study (equal to approx. 5.8 μg/ml when calculated as MW: 60 kDa). While the association of tissue inflammation and cancer progression has been reported [48, 49], future study should address a possible link regarding how chronic inflammation affects cancer cell behavior in vascular space.

The effect of sE-selectin was highly specific to CD44^+/high^ BCs; its effect was minor or undetectable in CD44^-/low^ BCs and CD44 K/D MDA-MB-231 BCs. CD44 (HCELL) [34], belongs to a family of cell-surface glycoproteins, and differential forms of CD44 (e.g., CD44s and CD44v) are expressed in a variety of human leukocytes and solid tumors. Despite its lack of intrinsic catalytic activity, CD44 plays a role in the activation of signaling pathways through association with intracellular and extracellular proteins. CD44 interacts with various soluble extracellular components or ECM. Ligand binding to CD44 has been associated with numerous cellular processes such as migration, adhesion, signaling, and proliferation [50]. For example, the interaction of CD44 and its ligand, hyaluronic acid (HA), results in proliferation of eosinophils [51], B-cells [52], T-cells [53], keratinocytes [54], and human melanoma cells [55]. In addition to HA, L-selectin and E-selectin are reported to be ligands for distinct glycoforms of CD44 or CD44v, respectively [56]. The interaction of CD44 with Src family protein kinase, Rho-kinase, Tiam 1, and Vav2 triggers intracellular activation of PI3K, Ras, and Rac1, which promote cell migration, proliferation, and survival [57–61]. Although mounting evidence suggests the involvement CD44 in tumor progression, the roles of CD44 as well as its utility as a biomarker for primary tumors remains controversial due to its dual functions as a tumor promoter or suppressor and altered expression in CD44v [37, 38]. However, recent studies highlighted the positive association of CD44^+^ CTCs with hematogenous metastasis. The presence of CD44^+^ CTCs is linked to bone metastasis [62] and correlates with poor prognosis in breast cancer [63, 64]. While the CD44 has been used as a stem cell marker, our data suggest that CD44 may have functional role in hematogenous metastasis in the circulation 1) as a ligand for endothelial E-selectin at the pre-metastatic niche [13] and 2) as a signaling molecule that mediates sE-selectin-induced extrinsic stimuli in circulating cells.

Abundant leukocyte infiltration into the tumor is associated with poor prognosis in many types of cancer including breast, prostate, lung cancer, and melanoma [48, 65, 66]. Our results demonstrate that sE-selectin enhancement of leukocyte migration is partially mediated by CD44. We used humanized NOD SCID IL2R-γ chain knockout mice, which are deficient in T-lymphocytes, B-lymphocytes, and NK cells, to recapitulate the effect of crosstalk between the human immune system and human cancer cells. Mice receiving human PBMCs treated with human sE-selectin showed rapid tumor growth, which coincided with increased intratumoral infiltration of PBMCs, in particular, CD4^+^ T-lymphocytes (Fig. 6c). Although we expected the CD4+/CD8+ balance shift to trigger pro-inflammatory signaling that would lead to an increase in the human CD68+ macrophage population, no change was noted in CD68+ cells or CD8+ T-lymphocytes. A separate study showed that sE-selectin-mediated tumor growth was not as significant when mouse splenocytes were infused into athymic nu/nu mice bearing xenograft tumors derived from MDA-MB-231 cells (data not shown), although infiltration of mouse PBMCs into the tumor was more extensive than that of human PBMCs (Additional file 1: Figure S6). Possibly, there is a slight discordance between adaptive immune cells from human and innate immune cells and vessels of mice.

**Fig. 7:**
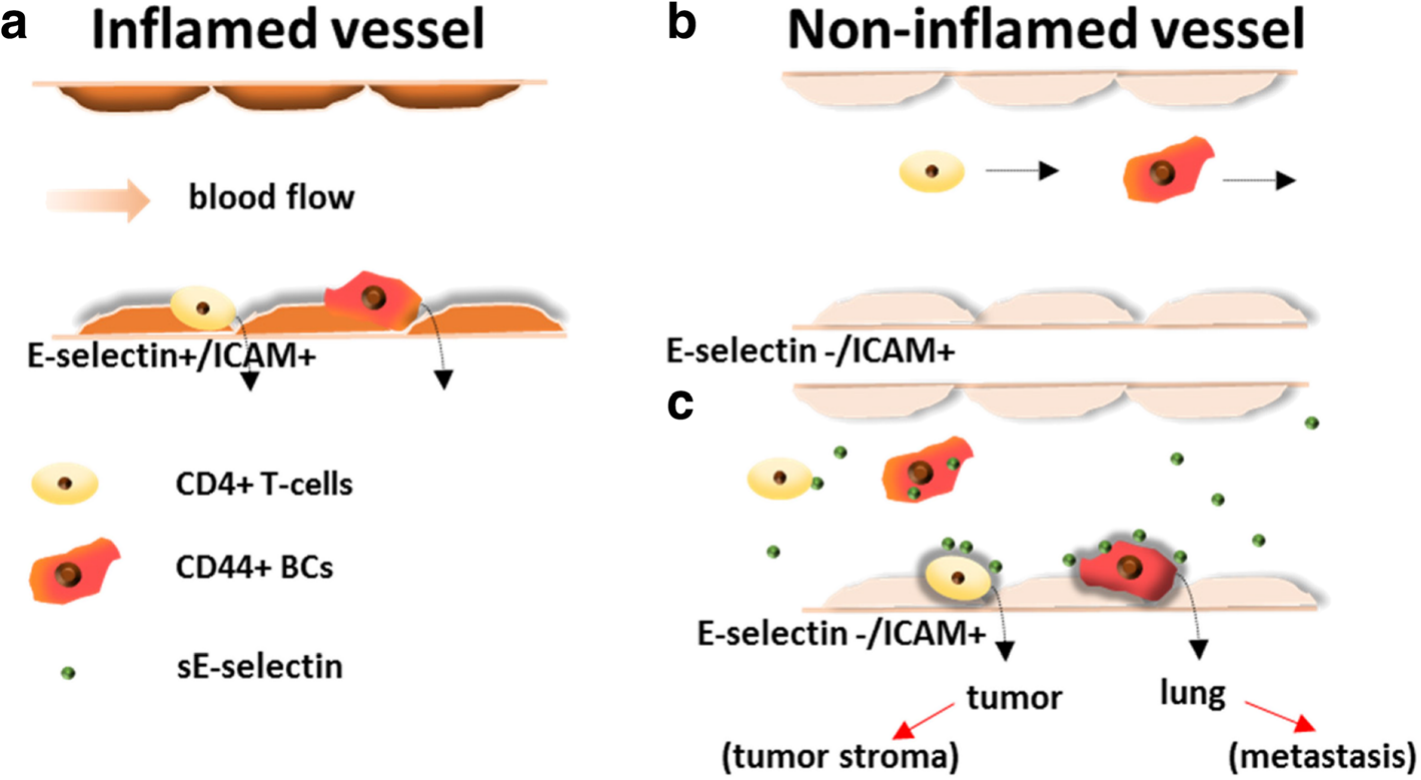
Scheme of sE-selectin-mediated shear-resistant adhesion and migration: **a** The expression of adhesion molecules such as E-selectin, ICAM, and VCAM is induced in the luminal surface of inflamed vessel. Margination of leukocytes and CD44^+/high^ BCs toward the vessel surface is followed by tethering, rolling, and adherence to inflamed vessels. The adhesion of the cells increases the permeability of endothelial junctions facilitating tissue migration of circulating cells. **b** By contrast, in a non-inflamed vessel, circulating cells remain in circulation and no random adhesion occurs. **c** Soluble E-selectin is shed from activated endothelium of inflamed tissues and activates both leukocytes and CD44^+/high^ BCs, which enhances their shear resistant adhesion to non-inflamed vessels and migration in the absence of E-selectin but via interaction with ICAM at low shear stress. Excessive infiltration of CD4^+^ T-lymphocytes into the tumor leads to development of pro-tumorigenic stroma. Infiltration of CD44^+/high^ BCs into distant organ increases the risk of colonization by metastatic cells

In conclusion, we proposed possible simultaneous and multifaceted effects of E-selectin in tumor growth and metastasis in Fig. 7: endothelial E-selectin mediates the shear-resistant adhesion of circulating tumor cells and immune cells to inflamed vessels (a), but not to non-inflamed vessels (b); sE-selectin shed into circulation accelerates homing of CD44^+^ BCs into tissue via non-inflamed vessels, endothelial E-selectin independent/ICAM dependent manner (c); sE-selectin accelerates the infiltration of CD4+ lymphocytes into the tumor to contribute to the development of inflammatory stromal (d).

## Conclusion

Soluble E-selectin (sE-selectin) is shed into the circulation from the activated endothelium. We investigated the effect of sE-selectin on shear-resistant adhesion and migration of metastatic breast cancer cells and leukocytes. Our data suggest that sE-selectin promotes migration and shear-resistant adhesion of circulating cells to non-activated HMVECs via CD44/FAK. This sE-selectin-mediated pleiotropic effects result in enhancement of homing of CD44^+/high^ BCs and immune cells to the lung and tumor, respectively. These findings identify a possible extrinsic mechanism by which serum factor stimulates circulating tumor cells toward tumor progression and metastasis.

## Additional files

Additional file 1:
**Supplementary Figure 1.** Soluble E-selectin enhances the anoikis resistance of ER-negative BCs: BCs were suspended (104 cells) in 1% FBS media with or without sE-selectin, and seeded in ultra-low attachment plates. The data represent the increased percentage of surviving cells with sE-selectin. The data shown are Mean ± SD. **Supplementary Figure 2.** The effect of FAK kinase inhibitors on cell viability: (a) HL-60 cells were incubated with the indicated concentrations of FAK inhibitor for 30 min. The viable cells were counted using a Neubauer hemocytometer 24 hours later. (b) MDA-MB-231 cells were incubated with the indicated concentrations of FAK inhibitor for 30 min. The cell viability was measured by MTT assay. **Supplementary Figure 3.** Profile of human PBMCs: PBMCs were isolated from freshly isolated human buffy coat of healthy donors. PBMCs were analyzed by flow cytometry using a FACS Calibur. **Supplementary Figure 4.** Detection of sE-selectin in the serum from mice bearing tumor: MDA-MB-231 cells (3x104 or 3x105 cells) were injected into mice (n=4). Two weeks later, whole blood was collected and serum was collected. Soluble E-selectin level was measured by ELISA. The data shown are Mean ± SD. **Supplementary Figure 5.** Soluble E-selectin enhances migration of MDA-MB-231 in sE-selectin dose dependent manner: Boyden chamber assay was performed. The migrated cells were stained using an HEMA3 staining kit and were counted under light microscopy. Data were expressed as percentage of control (saline treated as 100%). The data represent Mean ± S.D. **Supplementary Figure 6.** Mouse splenocyte infiltration into human tumor: Mouse splenocytes were isolated from Athymic nu/nu mice and labeled with Calcein AM (green). The labeled splenocytes (107 cells) were infused intravenously into mice bearing tumors derived from MDA-MB-231 BCs. Tumors were harvested and frozen sections were counterstained with Hoechst 33342 (blue) and visualized using a fluorescent microscope at ×200. (PPTX 4183 kb)

## Abbreviations

BCs: breast cancer cells
CAMs: cell adhesion molecules
CD11c-APC: allophycocyanin-conjugated CD11c antibody
CD14-PE: phycoerythrin-conjuated CD14 antibody
CD44 K/D: CD44 knock down
CD44: homing cell adhesion molecule (HCAM), phagocytic glycoprotein-1(Pgp-1)
CD44s: CD44 standard from
CD44sh: shRNA targeting CD44
CD44v: CD44 variant isoforms
CD45-FITC: fluorescein isothiocyanate-conjugated CD44 antibody
CD4-APC-eFluor780: allophycocyanin-eFluor780-conjugated CD4 antibody
CD68+: cluster of differentiation 68 positive
CD8-PerCPCy5.5: peridinin chlorophyll-cyanine5.5-conjugated antibody
CXCR4: Chemokine receptor 4
DMEM: Dulbecco’s modified eagle medium
dyn/cm2: dyne per one square centimeter
ER+: estrogen receptor positive
E-selectin: endothelial leukocyte adhesion molecule, CD62E
ESL-1: E-selectin ligand 1
FAK: Focal adhesion kinase
FITC: fluorescein isothiocyanate
GAPDH: glyceraldehyde-3-phosphate dehydrogenase
HCELL: hematopoietic cell E-selectin/L-selectin Ligand
HMVECs: human microvessel endothelial cells
ICAM: Intercellular adhesion molecules
Muc-1: Mucin 1
NSG mice: NOD scid gamma mice
PBMCs: peripheral blood mononuclear cells
pFAK: phosphorylated focal adhesion kinase
pHMVEC: parental HMVECs
PI3K: phosphoinositide 3-kinase
Rac1: ras-related C3 botulinum toxin substrate 1
Ras: rat sarcoma viral oncogene
SDF-1: stromal cell-derived factor 1
sE-selectin: soluble E-selectin
Src: proto-oncogene tyrosine kinase
VCAM: vascular cell adhesion molecules
VE-cadherin: vascular endothelial cadherin
VLA-4: very late antigen-4 (Integrin alpha4 beta1)
