# Erratum to: The effect of soluble E-selectin on tumor progression and metastasis

**DOI:** 10.1186/s12885-016-2391-1

**Published:** 2016-06-28

**Authors:** Shin-Ae Kang, Celine A. Blache, Sandra Bajana, Nafis Hasan, Mohamed Kamal, Yoshihiro Morita, Vineet Gupta, Bilegtsaikhan Tsolmon, K. Stephen Suh, David G. Gorenstein, Wajeeha Razaq, Hallgeir Rui, Takemi Tanaka

**Affiliations:** Department of Pathology, University of Oklahoma Health Sciences Center, 975 NE, 10th, Oklahoma City, OK 73104 USA; Thomas Jefferson University, Pharmaceutical Sciences, 1020 Locust St, Philadelphia, PA 19107 USA; John Theurer Cancer Center, Hackensack University Medical Center, Hackensack, NJ 07601 USA; Institute of Molecular Medicine, University of Texas Health Science Center at Houston, 1825 Hermann Pressler, Houston, TX 77030 USA; Department of Internal Medcine, Stephenson Cancer Center, University of Oklahoma Health Sciences Center, 800 NE, 10th, Oklahoma City, OK 73104 USA; Department of Pathology, Medical College of Wisconsin Cancer Center, 8701 Watertown Plank Rd, Milwaukee, WI 53226 USA; Stephenson Cancer Center at the University of Oklahoma Health Sciences Center, 975 NE 10th, Oklahoma City, OK 73104 USA

## Erratum

Unfortunately, the original version of this article [1] contained an error. The name of the author K. Stephen Suh was incorrectly written as Stephen K. Suh. The correct spelling is included here, and has been updated in the original article.
