# Supplementary material for: The effect of soluble E-selectin on tumor progression and metastasis
Source: BMC Cancer. 2016 May 24;16:331. doi: 10.1186/s12885-016-2366-2 (PMC4879723; doi:10.1186/s12885-016-2366-2)
Supplement: Additional file 1: — Supplementary Figure 1. Soluble E-selectin enhances the anoikis resistance of ER-negative BCs: BCs were suspended (104 cells) in 1% FBS media with or without sE-selectin, and seeded in ultra-low attachment plates. The data represent the increased percentage of surviving cells with sE-selectin. The data shown are Mean ± SD. Supplementary Figure 2. The effect of FAK kinase inhibitors on cell viability: (a) HL-60 cells were incubated with the indicated concentrations of FAK inhibitor for 30 min. The viable cells were counted using a Neubauer hemocytometer 24 hours later. (b) MDA-MB-231 cells were incubated with the indicated concentrations of FAK inhibitor for 30 min. The cell viability was measured by MTT assay. Supplementary Figure 3. Profile of human PBMCs: PBMCs were isolated from freshly isolated human buffy coat of healthy donors. PBMCs were analyzed by flow cytometry using a FACS Calibur. Supplementary Figure 4. Detection of sE-selectin in the serum from mice bearing tumor: MDA-MB-231 cells (3x10^4^ or 3x10^5^ cells) were injected into mice (n=4). Two weeks later, whole blood was collected and serum was collected. Soluble E-selectin level was measured by ELISA. The data shown are Mean ± SD. Supplementary Figure 5. Soluble E-selectin enhances migration of MDA-MB-231 in sE-selectin dose dependent manner: Boyden chamber assay was performed. The migrated cells were stained using an HEMA3 staining kit and were counted under light microscopy. Data were expressed as percentage of control (saline treated as 100%). The data represent Mean ± S.D. Supplementary Figure 6. Mouse splenocyte infiltration into human tumor: Mouse splenocytes were isolated from Athymic nu/nu mice and labeled with Calcein AM (green). The labeled splenocytes (107 cells) were infused intravenously into mice bearing tumors derived from MDA-MB-231 BCs. Tumors were harvested and frozen sections were counterstained with Hoechst 33342 (blue) and visualized using a fluorescent microscope at ×200. (PPTX 4183 k [file 12885_2016_2366_MOESM1_ESM.pptx]

## Slide 1
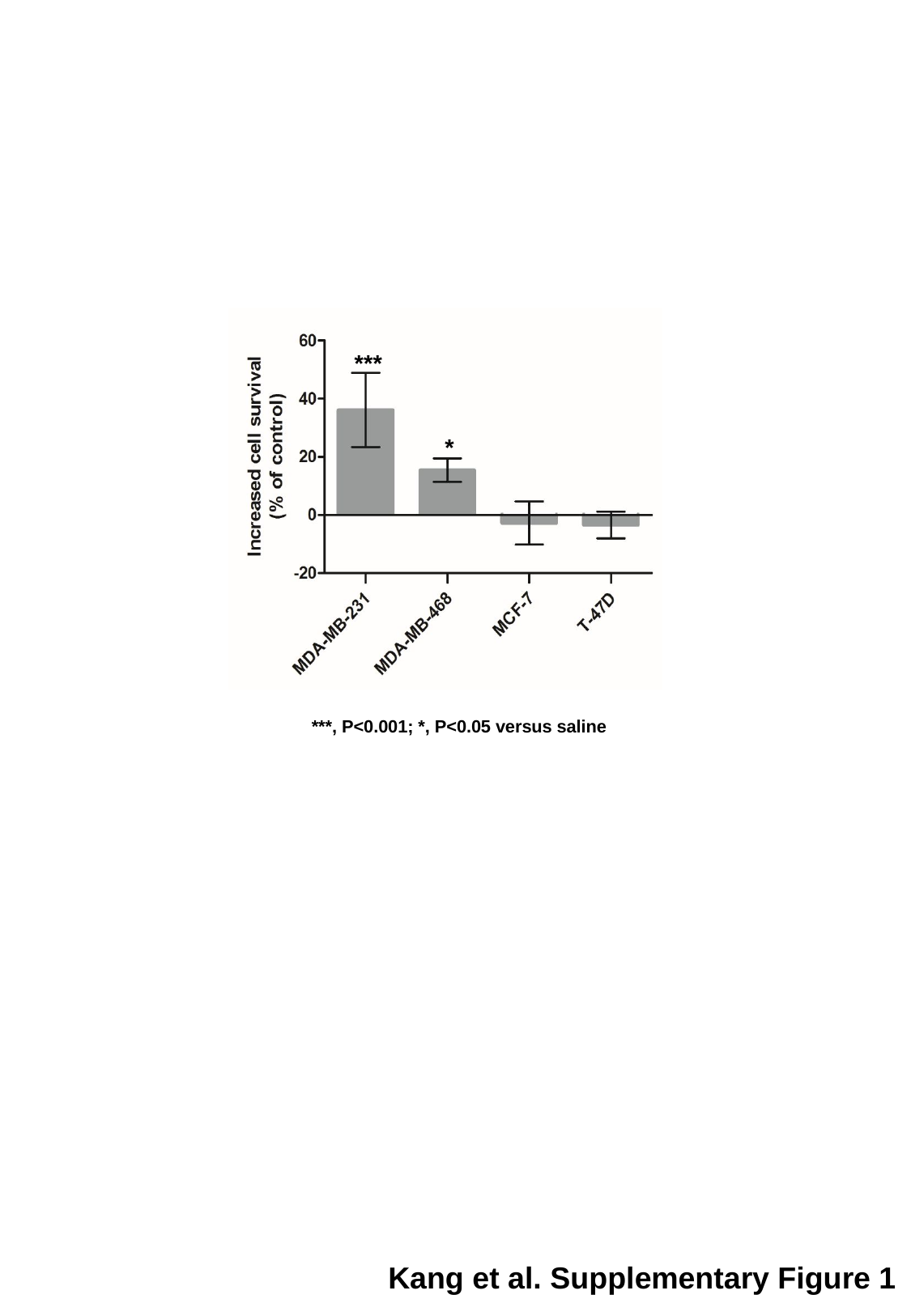

***
*
***, P<0.001; *, P<0.05 versus saline
Kang et al. Supplementary Figure 1

## Slide 2
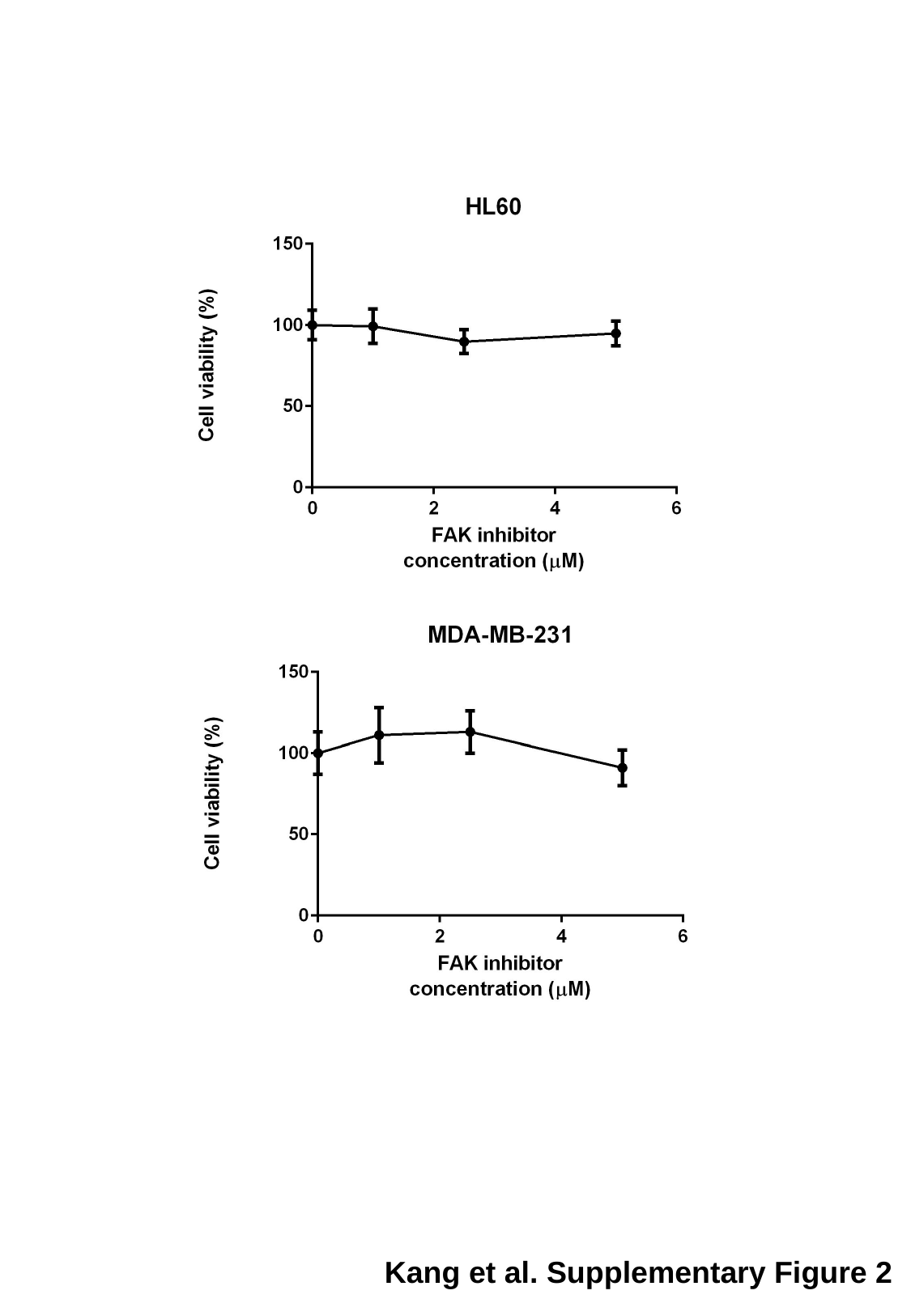

Kang et al. Supplementary Figure 2

## Slide 3
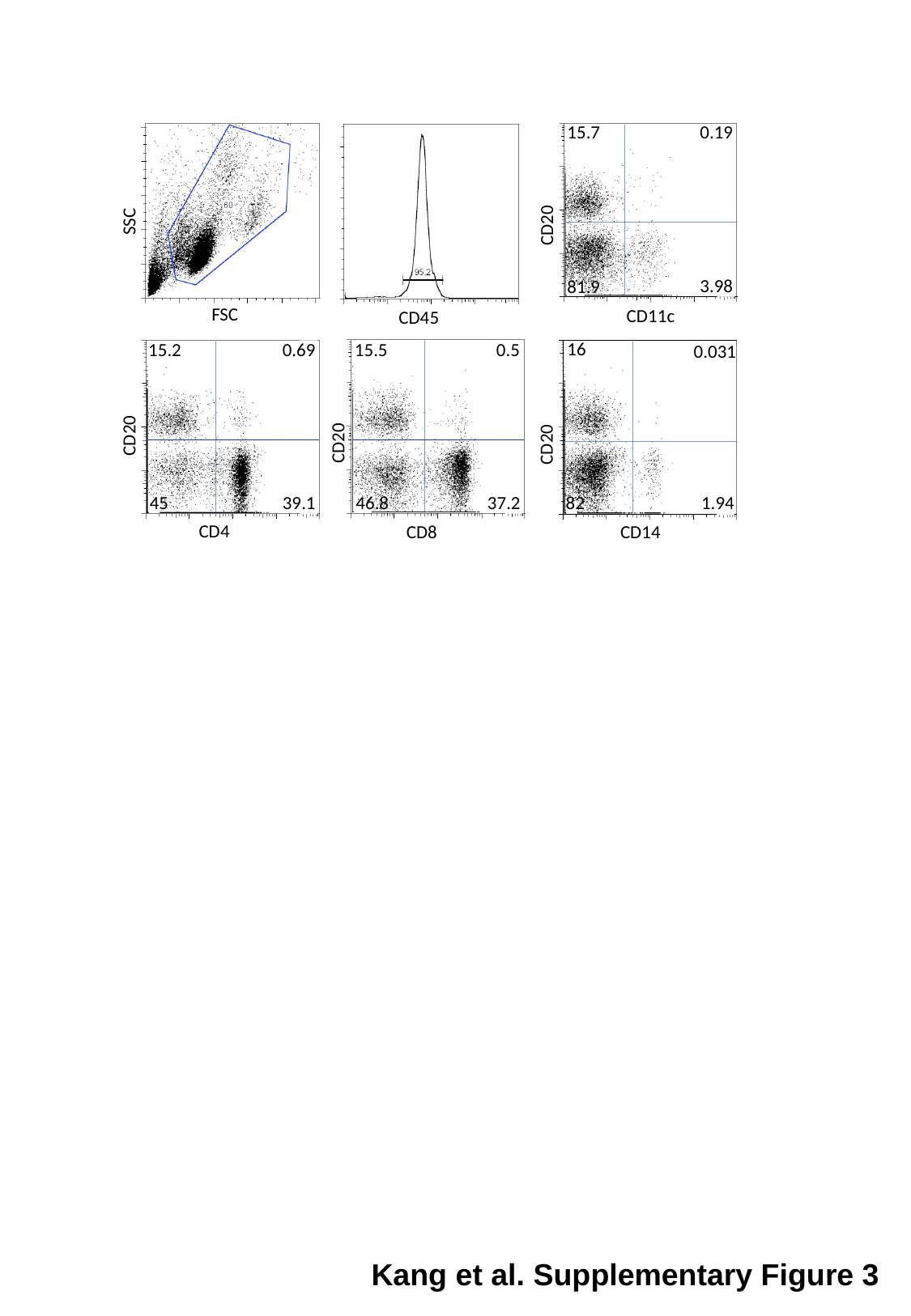

15.7
0.19
SSC
CD20
3.98
81.9
FSC
CD11c
CD45
16
15.2
0.69
15.5
0.5
0.031
CD20
CD20
CD20
45
39.1
46.8
37.2
82
1.94
CD4
CD8
CD14
Kang et al. Supplementary Figure 3

## Slide 4
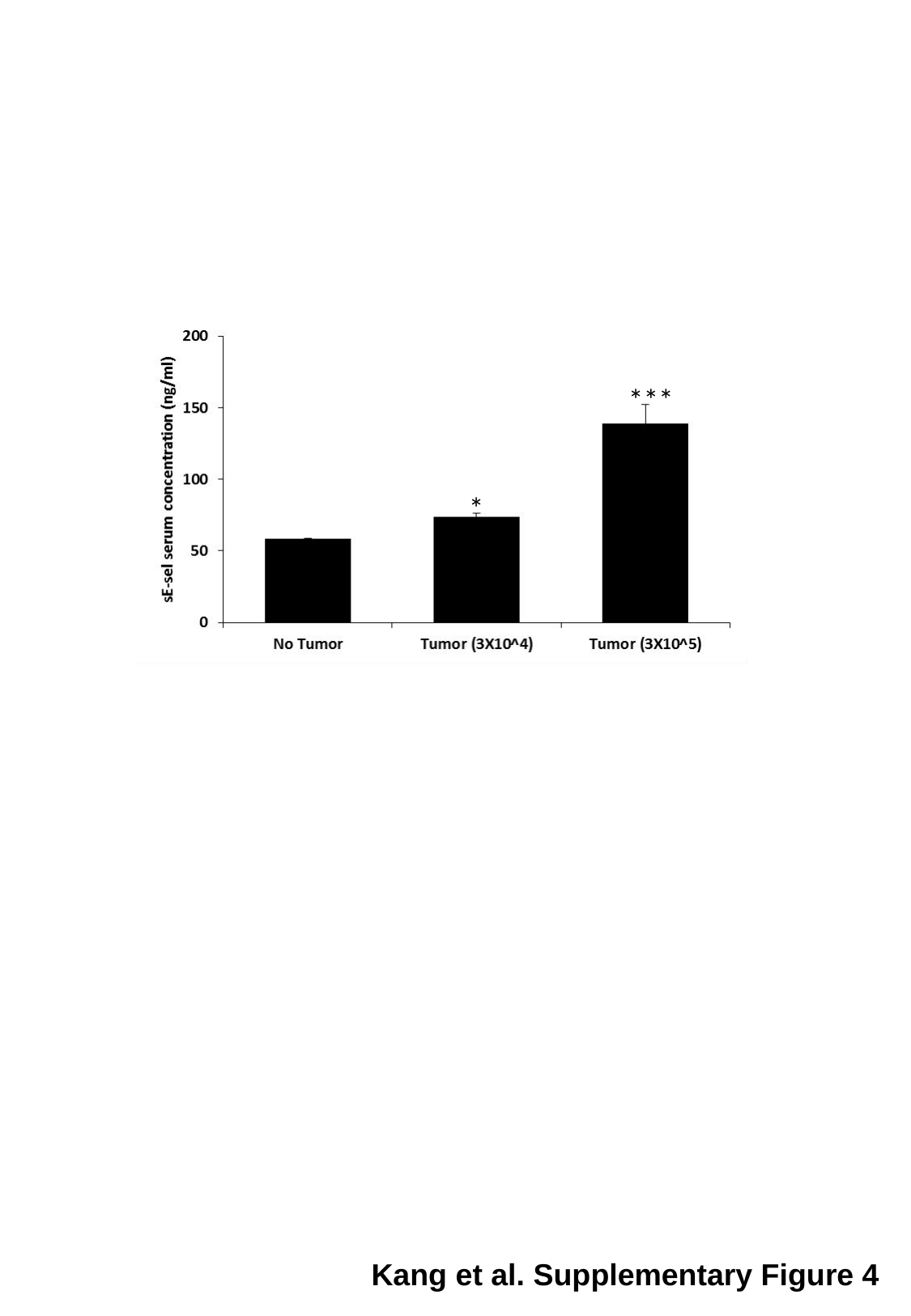

***
*
Kang et al. Supplementary Figure 4

## Slide 5
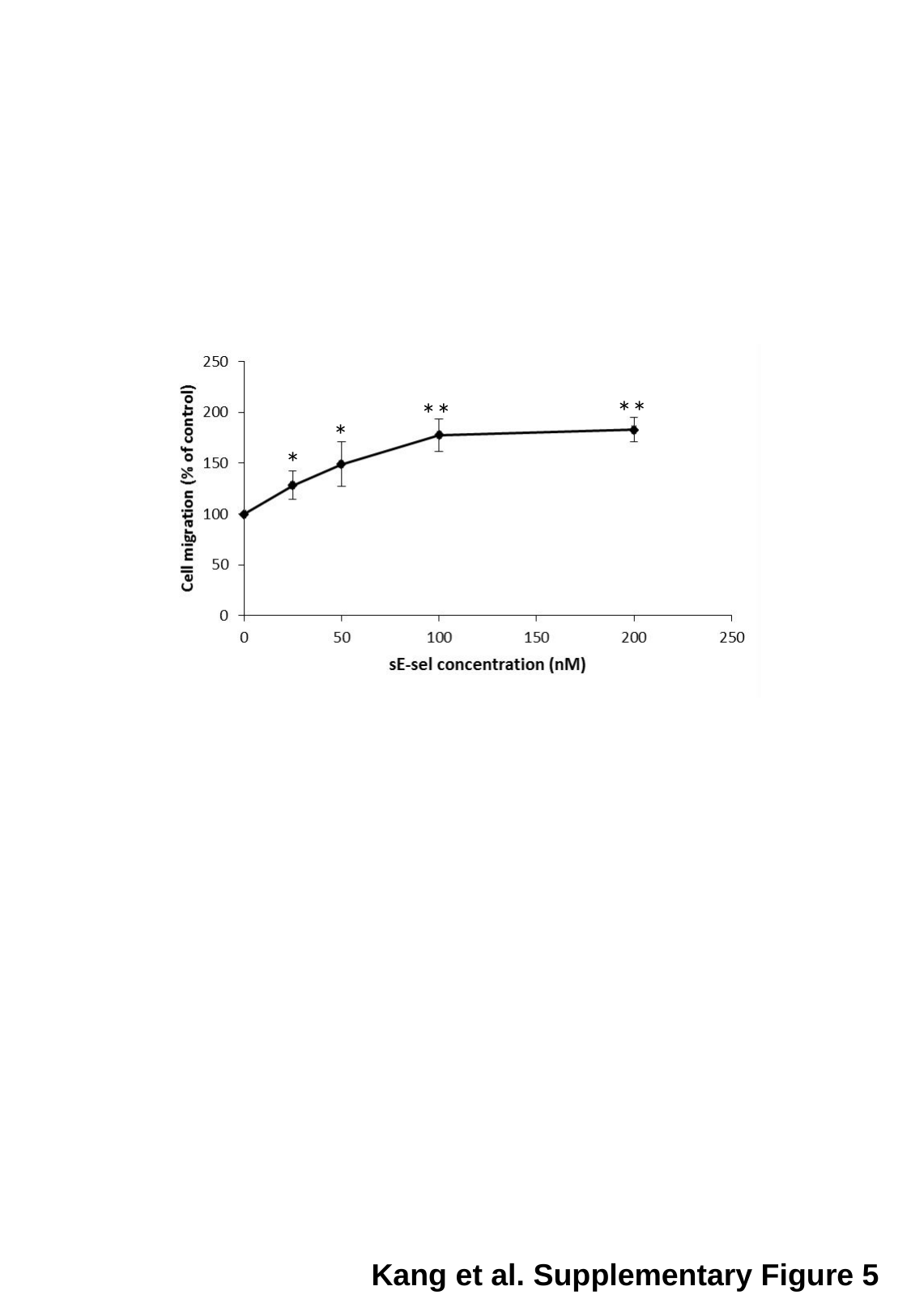

**
**
*
*
Kang et al. Supplementary Figure 5

## Slide 6
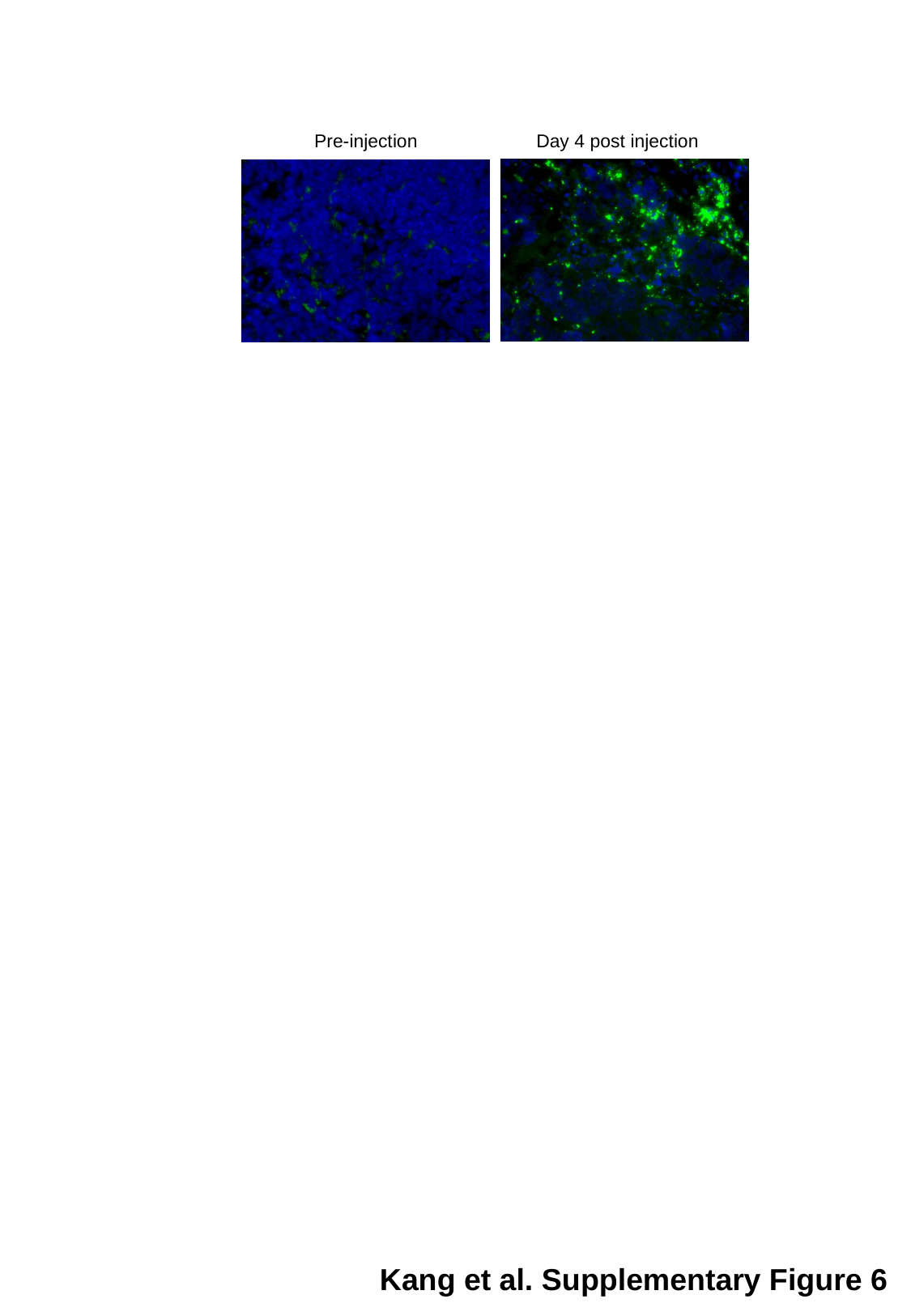

Pre-injection
Day 4 post injection
Kang et al. Supplementary Figure 6
